# Based On Confined Polymerization: In Situ Synthesis of PANI/PEEK Composite Film in One‐Step

**DOI:** 10.1002/advs.202103706

**Published:** 2021-11-11

**Authors:** Ziyu Lin, Ning Cao, Zhonghui Sun, Wenying Li, Yirong Sun, Haibo Zhang, Jinhui Pang, Zhenhua Jiang

**Affiliations:** ^1^ Key Laboratory of High Performance Plastics (Jilin University) Ministry of Education National & Local Joint Engineering Laboratory for Synthetic Technology of High Performance Polymer College of Chemistry Jilin University Jilin University Changchun 130012 P. R. China

**Keywords:** confined polymerization, dielectric, polyaniline, polyether ether ketone, polymer crystallization

## Abstract

Confined polymerization is an effective method for precise synthesis, which can further control the micro‐nano structure inside the composite material. Polyaniline (PANI)‐based composites are usually prepared by blending and original growth methods. However, due to the strong rigidity and hydrogen bonding of PANI, the content of PANI composites is low and easy to agglomerate. Here, based on confined polymerization, it is reported that polyaniline /polyether ether ketone (PANI/PEEK) film with high PANI content is synthesized in situ by a one‐step method. The micro‐nano structure of the two polymers in the confined space is further explored and it is found that PANI grows in the free volume of the PEEK chain, making the arrangement of the PEEK chain more orderly. Under the best experimental conditions, the prepared 16 µm‐PANI/PEEK film has a dielectric constant of 205.4 (dielectric loss 0.401), the 75 µm‐PANI/PEEK film has a conductivity of 3.01×10^−4^ S m^−1^. The prepared PANI/PEEK composite film can be further used as electronic packaging materials, conductive materials, and other fields, which has potential application prospects in anti‐static, electromagnetic shielding materials, corrosion resistance, and other fields.

## Graphical abstract

Polyaniline/polyether ether ketone (PANI/PEEK) composite film is successfully synthesized using PEEKt film as a template through confined polymerization. PANI and PEEK are co‐grown in situ by one‐step method, and the micro‐nano structure changes are further investigated. PANI/PEEK film has a significant application effect in high dielectric materials, conductive materials and other fields.

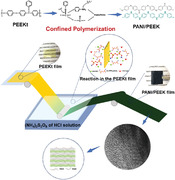

## 1 Introduction

Composites have been widely used in production and life because of their multiple advantages, and they are usually prepared by blending and in situ growth.^[^ ^1^, ^2^, ^3^, ^4^, ^5^ ^]^ Physical blending and chemical in situ growth are usually unable to further control the internal micro‐nano structure of the material. Confined polymerization is an important method for precise synthesis,^[^ ^6^, ^7^, ^8^, ^9^ ^]^ which can achieve precise control of the internal micro‐nano structure of the material.^[^ ^10^, ^11^, ^12^, ^13^, ^14^ ^]^ Perego et al. generated multiple anions on the designed framework, carried and removed protons at selected positions, and initiated chain propagation, realizing polymerization within the confined framework, which results in the chain being covalently connected to the 3D network.^[^ ^15^ ^]^ Wang's research group reported a strategy for preparing molecular sieve films. By confined polymerization of multifunctional rigid‐building units, the channels in the water ultrafiltration film (PSU) were divided into ultra‐micropores to realize a variety of gas separation films.^[^ ^16^ ^]^ Qiao's research group realized the fine control of carbon microspheres through the internal confinement cross‐linking of nitrogen‐doped multi‐chamber carbon (MCC) microspheres and the surfactant‐oriented space‐confined polymerization strategy.^[^ ^17^ ^]^

Conductive polymers have been widely used in composite fillers due to their unique conductive properties.^[^ ^18^, ^19^, ^20^ ^]^ Polyaniline (PANI) is one of the widely used conductive polymers, which is a new type of material with special functions. PANI has good thermal and chemical stability, which has the advantages of excellent environmental stability, high electrical conductivity, lightweight, easy processing, and cheap raw materials.^[^ ^21^, ^22^, ^23^ ^]^ PANI was first discovered in 1862, but researchers did not find that PANI had excellent electrochemical properties. It was not until the 1980s that MacDiarmid and others prepared PANI with conductivity in an acidic aqueous solution for the first time.^[^ ^24^, ^25^ ^]^ The preparation of PANI is usually carried out by electrochemical polymerization, oxidative polymerization, etc. The common oxidants are (NH_4_)_2_S_2_O_8_, K_2_CrO_7_, FeCl_3_, H_2_O_2_, and so on.^[^ ^26^, ^27^, ^28^, ^29^ ^]^ In 1987, MacDiarmid et al. finally proposed a widely recognized eigenstate PANI model, which contains two structural units, oxidation, and reduction. They found that the oxidation state of PANI depends on the ratio of benzene and quinone structures in its structure. What's more, different oxidation states can be transformed into each other.^[^ ^30^, ^31^ ^]^ As p‐type conductive polymer, PANI exhibits a variety of oxidation states during the conversion process, which shows a variety of colors. Its “neutral state” is yellow, “partially oxidized polyaniline salt” is green, and “partially oxidized polyaniline” is blue, “complete oxidation state” is purple.^[^ ^32^ ^]^ The intrinsic state PANI is basically close to the insulator, and its conductivity is very poor. Through proton doping and gradually changing the doping degree, an obvious transition of insulator‐semiconductor‐metal state can be realized. In the process of acid doping, HCl is the most commonly used doping acid. Commonly used organic acids are 4‐Dodecylbenzenesulfonic acid, Camphor‐10‐sulfonic acid, and Naphthalenesulfonic acid. Commonly used inorganic acids are HBr, H_2_SO_4_, HNO_3_, etc.^[^ ^33^, ^34^, ^35^, ^36^, ^37^ ^]^

As an outstanding conductive material, PANI is widely used in high dielectric materials.^[^ ^38^, ^39^ ^]^ The preparation of conductive filler/polymer composites is a significant method to obtain ultra‐high dielectric constant, which could be used in electronic packaging materials, embedded capacitors, significant application, etc.^[^ ^40^, ^41^ ^]^ SHEN et al. prepared composites of PANI fiber and PVDF copolymer, and changed the doping degree of PANI by exploring different acid doping. When the doping amount is 2.9%, the dielectric constant reaches 700.^[^ ^42^ ^]^ LEE and Rubinger have prepared PANI, polyacrylic acid (PAA), and polyvinylsulfonic acid (PSS) composites, and prepared high dielectric constant materials.^[^ ^43^, ^44^ ^]^ Wang et al. grafted the aniline oligomer to the end of the polyvinylidene fluoride‐trifluoroethylene copolymer by grafting, and explored the influence of different grafting amounts, and the dielectric constant of the prepared material was up to 85.^[^ ^45^ ^]^ SHAO et al. introduced a high‐permittivity permeable composite material, by oxidative coupling polymerization to graft oligoaniline (OANI) onto the acrylic resin elastomer (AE) chain (AE‐g‐OANI), the dielectric constant was 168, the dielectric loss was 0.21.^[^ ^46^ ^]^ WU et al. prepared acrylic resin elastomer (AR), chemically modified PANI (HBSiPA), and thermally reduced graphene oxide (TrGOs) ternary composites through free radical polymerization, and used PANI segments to enrich the electricity performance of graphene.^[^ ^38^ ^]^

With the further development of special engineering plastics, making full use of the advantages of polyether ether ketone (PEEK) polymers with high‐temperature resistance and good mechanical properties, the use of PEEK to prepare high dielectric materials has become a hot research topic. Because of its good performance, PANI‐based polyaryl ether ketone (PAEK) materials have potential application prospects in the fields of dielectric, conductive, electronic packaging materials, anti‐static, electromagnetic shielding materials, and corrosion resistance. The use of PAEK polymers and PANI to prepare high dielectric constant materials has been fully studied. Zhang et al. prepared PANI/SPAEK composite film by a solution blending method. Using the advantages of PAEK, the composite film with a dielectric constant of 600 and a dielectric loss of 0.6 was obtained, and the tensile strength reached 35MPa.^[^ ^47^ ^]^ Zhang et al. used in‐situ polymerization to prepare acidified multi‐walled carbon nanotubes (a‐MWCNTs) coated PANI (a‐MWCNTs@PANI) nanofillers and sulfonated polyaryl ether ketone (SPEEK) substrate dielectric infiltration composites.^[^ ^39^ ^]^ The dielectric constant is higher than 800. However, based on the composites of PANI conductive filler, PANI is very fragile due to its strong rigidity, and it is difficult to form a film. What‘s more, there are hydrogen bonds between the main chains of PANI molecules. The hydrogen bonds cause strong agglomeration between PANI molecular chains, and the solubility is very poor, which makes it difficult for PANI to form a film on its own and has a small content in the composites film.

**Figure 1:**
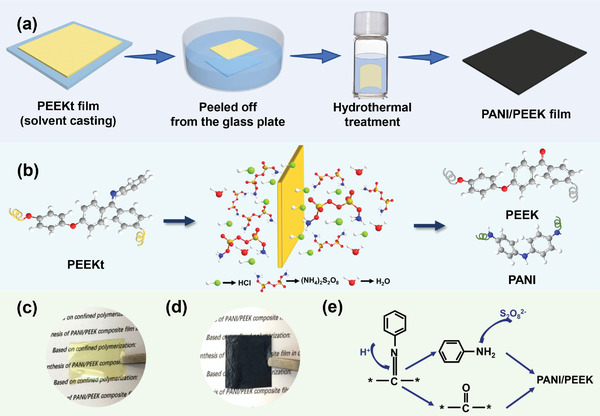
a) Schematic illustration of the method for PANI/PEEK composite films formation. b) Schematic diagram of the reaction in the film. c) Physical image of PEEKt film. d) Physical image of PANI/PEEK film. e) The reaction in the film.

Herein, we first reported the polyaniline / polyether ether ketone (PANI/PEEK) film was prepared in situ and in one‐step by confined polymerization through the template of PEEKt film, which realized the co‐growth of the two polymers in the confined space. (**Figure** **1**) The prepared PANI/PEEK film solves the problem that PEEK is difficult to form a film and PANI agglomerates, which achieves the high content of PANI of more than 15%. In addition, we further explored the changes in the micro‐nano structure inside the composite film during the formation process. The dielectric and conductive properties of the prepared PANI/PEEK composite film have been greatly improved, and the mechanical strength has been significantly enhanced. It has significant application prospects for further use as an electronic packaging material.

## 2 Results and Discussion

### 2.1 Characterization and Growth of the PANI/PEEK Film

The PANI/PEEK composite film is made by HCl and (NH_4_)_2_S_2_O_8_ entering the inside of the PEEKt film, which results in the aniline being confined polymerized to synthesize PANI. PANI and PEEK co‐grow in situ in the confined space, and finally prepare high PANI content of PANI/PEEK film. PEEKt is prepared from N‐phenyl (4,4′‐difluorodiphenyl) ketamine monomer and hydroquinone monomer through nucleophilic polycondensation. The specific synthesis route is shown in Schemes S1 and S2, Supporting Information.^[^ ^48^, ^49^ ^]^ Obtained by NMR analysis comparing all the protons of PEEKt and monomer accurately, two obvious new peaks appeared at 6.71 and 6.73 ppm, which were attributed to the H^a^ on the benzene ring, indicating that the preparation of PEEKt was successful. (Figures S1 and S2, Supporting Information) NMR characterization strongly illustrates the successful preparation of PEEKt polymer.^[^ ^50^, ^51^ ^]^ In the previous experiments of our research group, it was proved that the polymer precursor (PEEKt) can be hydrolyzed by HCl solution to realize the complete conversion of PEEKt to PEEK.^[^ ^48^, ^49^, ^52^ ^]^ Based on previous work, the PANI/PEEK composite film was prepared by the method of confined polymerization, using PEEKt film as the template, and the one‐step method of confined polymerization within the homogeneous film by means of hydrolysis and oxidation.

**Figure 2:**
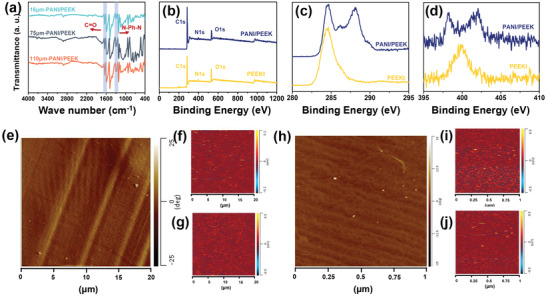
a) ATR‐FTIR spectrum of PANI/PEEK films. Deconvoluted XPS spectra: b) The survey spectra of PEEKt and PANI/PEEK films; XPS core spectra of c) C 1s, and d) N 1s of PEEKt and PANI/PEEK films. Nano IR of PANI/PEEK films: e) AFM‐Phase image, f) Nano IR, Absorption peak at 1650 cm^−1^, g) Nano IR, Absorption peak at 1306 cm^−1^. Scale bar was 20 µm. h) AFM‐Phase image, i) Nano IR, Absorption peak at 1650 cm^−1^, j) Nano IR, Absorption peak at 1306 cm^−1^. Scale bar was 1 µm.

As shown in **Figure** **2**a, the infrared spectrum confirmed the successful preparation of the PANI/PEEK composite films. Under the influence of HCl, Schiff alkali undergoes hydrolysis, and the ketimine is converted into ketone, forming PEEK chain. After processing, the infrared spectra of PANI/PEEK films of different thicknesses all showed a C═O vibration peak at 1650 cm^−1^, which proved the successful preparation of PEEK. Under the influence of (NH_4_)_2_S_2_O_8_, the aniline monomer that is hydrolyzed off will attack the para‐electron of aniline, and the aniline will self‐polymerize inside the film to form PANI chain. It can be seen from the infrared spectrum that the C—N vibration peak for N—Ph—N appears at 1306 cm^−1^ of the PANI/PEEK film.^[^ ^52^, ^53^, ^54^, ^55^ ^]^ As shown in Figure S3, Supporting Information, the film treated with a single hydrochloric acid can only become PEEK, and the group of the film treated with a single oxidation has not changed, which shows that the synergistic effect of hydrochloric acid and ammonium persulfate promotes the formation of the PANI/PEEK composite film.

In order to further verify the specific position of PANI in the polymerization process, we used XPS to further characterize the surface of the composite film and the inner part of the film by Nano‐IR. As shown in Figure 2b, the survey spectrum of PEEKt and PANI/PEEK films contains three elements: C, N, and O. It can be seen from the position of the PANI/PEEK film in the N spectrum in Figure 2d that the N1s spectrum is smoothly fitted to four sub‐peaks. The 398.1 eV peak is related to the undoped imine unit. The peak of 399.1 eV has the same energy as the previously reported N 1s of undoped amine unit. The 400.9 eV peak is related to the cationic nitrogen atom (polaron and dipolaron). The 402.6 eV peak is related to the protonated amine units.^[^ ^56^, ^57^, ^58^ ^]^ These protonated amine units have higher binding energy. This is due to the conjugation at the sp^3^ bond site leading to a stronger electronic response. The test shows that the aniline inside the film diffuses to the film surface and the aniline that falls off the film surface polymerizes on the film surface to obtain PANI.

The PANI/PEEK composite film was sliced at room temperature using an ultra‐thin microtome, and the sliced film was subjected to Nano‐Infrared analysis of the cross‐section film. The infrared spectra of the film cross‐section showed an N—Ph—N peak at 1306 cm^−1^ and a C═O peak at 1650 cm^−1^, which strongly proved that PANI was growing within the film. (Figures S4 and S5, Supporting Information) As shown in Figure 2f,g, the nano‐infrared image at 1306 cm^−1^ shows that the PANI is uniformly distributed inside the film without agglomeration. With regard to the small‐scale analysis, it shows that PANI is well dispersed inside the film, and there is no obvious interface with the characteristic peak of PEEK (absorption peak at 1650 cm^−1^). (Figure 2i,j)^[^ ^59^, ^60^ ^]^ The Nano‐IR shows that PANI and PEEK are interlaced with each other, and no phase separation occurs. During the growth process of PANI, it grows in the free volume of PEEK. The presence of PEEK chains effectively hinders the agglomeration of PANI and prevents PANI from agglomeration caused by excessive hydrogen bonding. The use of the in‐situ confined polymerization method effectively increases the content of PANI. Through the method of weight loss, the content of PANI measured by the experiment is above 15% and can reach 20%. (Figure S6, Supporting Information) At the same time, through the element content test (Table S1, Supporting Information), the O element has further increased, which further shows that the PEEK is produced, and the loss of N element is not much, which shows that the high content of PANI has been synthesized. This confined polymerization method effectively realizes the effective combination of rigid/insoluble polymers, and at the same time, according to the analysis of Nano‐Infrared, it once again confirms the uniform dispersion of PANI and PEEK, and achieves a high content of PANI.

### 2.2 Growth Mechanism of the PANI/PEEK Film

**Figure 3:**
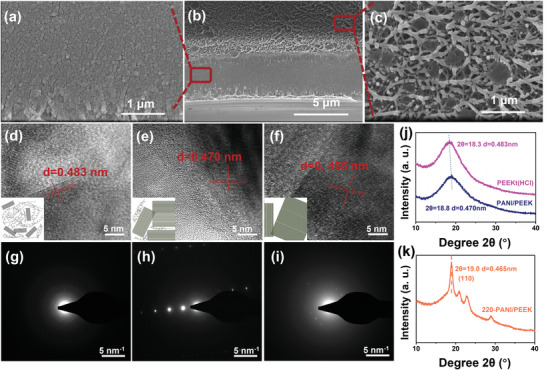
a–c) The cross‐sectional SEM images of 75 µm‐PANI/PEEK films. HRTEM of the as‐prepared films: d) PEEK film (PEEKt treated with HCl); e) 75 µm‐PANI/PEEK film. f) 220‐PANI/PEEK (The PANI/PEEK film was annealed at 220 °C). SAED of the as‐prepared films: g) PEEK film (PEEKt treated with HCl); h) 75 µm‐PANI/PEEK film. i) 220‐PANI/PEEK (The PANI/PEEK film was annealed at 220 °C). The inset images are the illustration of the distribution of the lattice. XRD of j) PEEKt film treated with HCl and PANI/PEEK film. k) 220‐PANI/PEEK (The PANI/PEEK film was annealed at 220 °C).

In order to in‐depth study the formation of composites, as well as the internal formation mechanism of PANI/PEEK film. The film formation method was confirmed by SEM and HRTEM. During the growth process, it is divided into three parts (Figure S7, Supporting Information). In the first part, the film surface first contacts the solution, and the ketimine structure on the film surface first contacts hydrochloric acid, and hydrolyzes to form aniline and PEEK. In contact with the oxidant, a small part of the aniline diffuses into the solution, forming PANI in the solution, and part of the aniline polymerizes in‐situ on the film surface to form PANI. The second part, as the HCl and (NH_4_)_2_S_2_O_8_ gradually deepens from the film surface to the inside of the film, small pores are formed on the film surface to promote hydrochloric acid. (Figure S7e, Supporting Information, **Figure** **3**a,b) Besides, the entry of (NH_4_)_2_S_2_O_8_ forms a loose thin layer on the side close to the surface of the film, the diffusion of aniline monomer is hindered, a small part of aniline diffuses into the solution, and most of the aniline is polymerized in the film to form PANI. The third part, as the HCl and (NH_4_)_2_S_2_O_8_ enter the middle to the inside of the film, the layered grid structure is gradually formed in the film (Figure 3b,c). The hydrolysis of amine prevents the diffusion of aniline in the film, combines with HCl and (NH_4_)_2_S_2_O_8_, and polymerizes in‐situ in the film to form PANI. Finally, the PANI/PEEK composite film is formed, and the PANI grows in‐situ inside the film. The AFM image of the film shows the striped internal structure, which is consistent with the cross‐sectional view of the SEM, indicating that a special network structure is formed inside the film. (Figure 2e,h) The confined polymerization inside the film further restricts the movement of the molecular chain and forms a special network structure.

The nitrogen adsorption test was used to specify the structure of the pores. In the process of forming, the network structure and microporous structure will appear inside the film. (Figure 3a–c) Based on nitrogen adsorption, BET surface areas of the films were calculated.^[^ ^17^ ^]^ The BET of the 75 µm‐PANI/PEEK film is 223.71 m^2^ g^−1^ and the BET of the 16 µm‐PANI/PEEK film is 429.82 m^2^ g^−1^. (Figure S8, Supporting Information) When the thickness of the PEEKt film increases, molecular diffusion is hindered, which causes that it is easier for the 16 µm‐PANI/PEEK film to diffuse compared to the 75 µm‐PANI/PEEK film. It can be seen from the pore size distribution that the pore size of the 16 µm‐PANI/PEEK film is concentrated at 3 nm, while the pore size of the 75 µm‐PANI/PEEK film is wider at 3–7 nm. (Figure S8a,d, Supporting Information) This further shows that due to the hindered film diffusion, the film has fewer pores but a wider distribution. When the film is thinner, more pores are formed and the pore size distribution is narrower.

Based on the formation of the special network structure, we deeply explored the changes in the micro‐nano structure inside the film, using XRD, DMA, HRTEM, and SAED to analyze the micro‐nano changes of PANI and PEEK during the formation of the film. After the hydrochloric acid treatment, polymer crystals appeared, the crystallization behavior was incomplete, the intermittent lattice structure appeared, and the crystal lattice was discontinuous. (Figure 3d) In the process of confined polymerization, PANI restricted the movement of the PEEK chain during the formation process, and finally forms PANI/PEEK with better crystallinity and continuous lattice stripes composite materials. (Figure 3e)^[^ ^61^, ^62^, ^63^ ^]^ By comparison (Figure 3j) after the oxidation treatment, the addition of PANI increased the 2*θ* angle (2*θ* changed from 18.3° to 18.8°). According to the **Formula S1**, Supporting information, the interlayer spacing was reduced (d changed from 0.483 nm to 0.470 nm), indicating that during the growth process, PANI grew in the free volume between the chains of PEEK.^[^ ^64^, ^65^ ^]^ Through the *T*_g_ of the DMA test (Figure S9, Supporting Information), it can be seen that the *T*_g_ of the treated film decreases (*T*_g_ decreased from 130.1 ^o^C to 127.8 °C), indicating that the introduction of PANI chains increases the chain spacing of the PEEK and weakens the rigidity, so the *T*_g_ decreases.^[^ ^15^, ^66^ ^]^ In order to further verify the influence of PANI on the PEEK chain, the film was annealed at 220 °C. As shown in Figure 3f,k, the crystallinity of the film after annealing is enhanced, and the crystallization peak of PEEK appears, besides, the *T*_g_ (*T*_g_ = 172 °C) is significantly higher than the *T*_g_ of the commercial PEEK film (*T*_g_ = 150 °C). Because during the annealing process, the PEEK chain restricted by the chain of PANI, the movement of the PEEK chain is hindered. At the same time, the rigidity of PANI is relatively strong, so the *T*_g_ of the composite material increases significantly after annealing. Through the HRTEM, it can be seen that the lattice spacing of the annealed film is reduced, the crystal peak is stronger, and the crystal lattice is more continuous. According to SAED, there are electronic bright spots in all three films, and they all have certain crystallization behavior. (Figure 3g–i)^[^ ^67^ ^]^ As shown in Table S2, Supporting Information, the PEEKt (HCl) film has a reduced density due to the aniline shedding density of 1.20 compared to the commercial PEEK film density of 1.28. The film after confined polymerization increases the density of the film due to the presence of PANI. During the annealing process, the movement of molecular chains results that the molecular packing becomes more regular, and the density increases to 1.29. This shows that PANI will grow in the free volume around the PEEK chain during the growth process, and PANI will further confine the movement of the PEEK chain at the same time.

### 2.3 The Influence of Different Conditions on PANI/PEEK Film Formation

In order to further study the influence of different conditions on the film formation, the films were fabricated with different conditions of temperature, c(HCl), and c((NH_4_)_2_S_2_O_8_) and their performance (the dielectric, conductive properties, and microscopic morphology) were studied. On the samples with parallel plate capacitor configuration, the conductivity and dielectric properties of PANI/PEEK composites under different conditions were studied. In the experiment to explore the temperature change, due to the polarization of the interface and space charge, the dielectric constant and the conductivity first increased and then decreased with the change of frequency, and the maximum value appeared at 60 °C. (Figures S10 and S11, Supporting Information) As the temperature increases, the surface particles gradually aggregate and increase, at the same time, small pores and PANI particles appear inside the film, and the pores gradually become larger. (Figure S12, Supporting Information) This shows that temperature promotes the formation of PANI, but too high temperature causes the aniline to diffuse faster and the morphology of PANI changes. Similarly, the films treated with different c(HCl) also showed the same trend of change, reaching the maximum at 2.5 mol L^−1^ (Figures S13 and S14, Supporting Information), which is because during the treatment process the particles on the surface of the film gradually increase, and micropores appear on the surface. (Figure S15, Supporting Information) It can be seen that the increase of c(HCl) will promote the formation of a network structure inside the film and promote the diffusion of aniline. When the c((NH_4_)_2_S_2_O_8_) inside the film increases, the particles inside the film become larger and more numerous, which resulted in the dielectric constant and electrical conductivity first increasing and then decreasing. (Figures S16–S18, Supporting Information) This proves that the increase of c((NH_4_)_2_S_2_O_8_) will further promote the formation of PANI particles. Samples under all conditions show a decrease in frequency as the frequency increases. The sample has a stronger frequency dependence, and the peaks appearing at high K values may be related to the interface polarization relaxation effect.^[^ ^38^ ^]^ Based on the above experimental data, we found the best experimental conditions are the temperature at 60 °C, 2.5 mol L^−1^ at c(HCl), and 0.037 mol L^−1^ at c((NH_4_)_2_S_2_O_8_). The exploration of the above conditions provides a basis for the subsequent preparation, which can further control the thickness of the film to prepare dielectric and conductive materials.

### 2.4 Dielectric and Conductive Properties of PANI/PEEK Film

**Figure 4:**
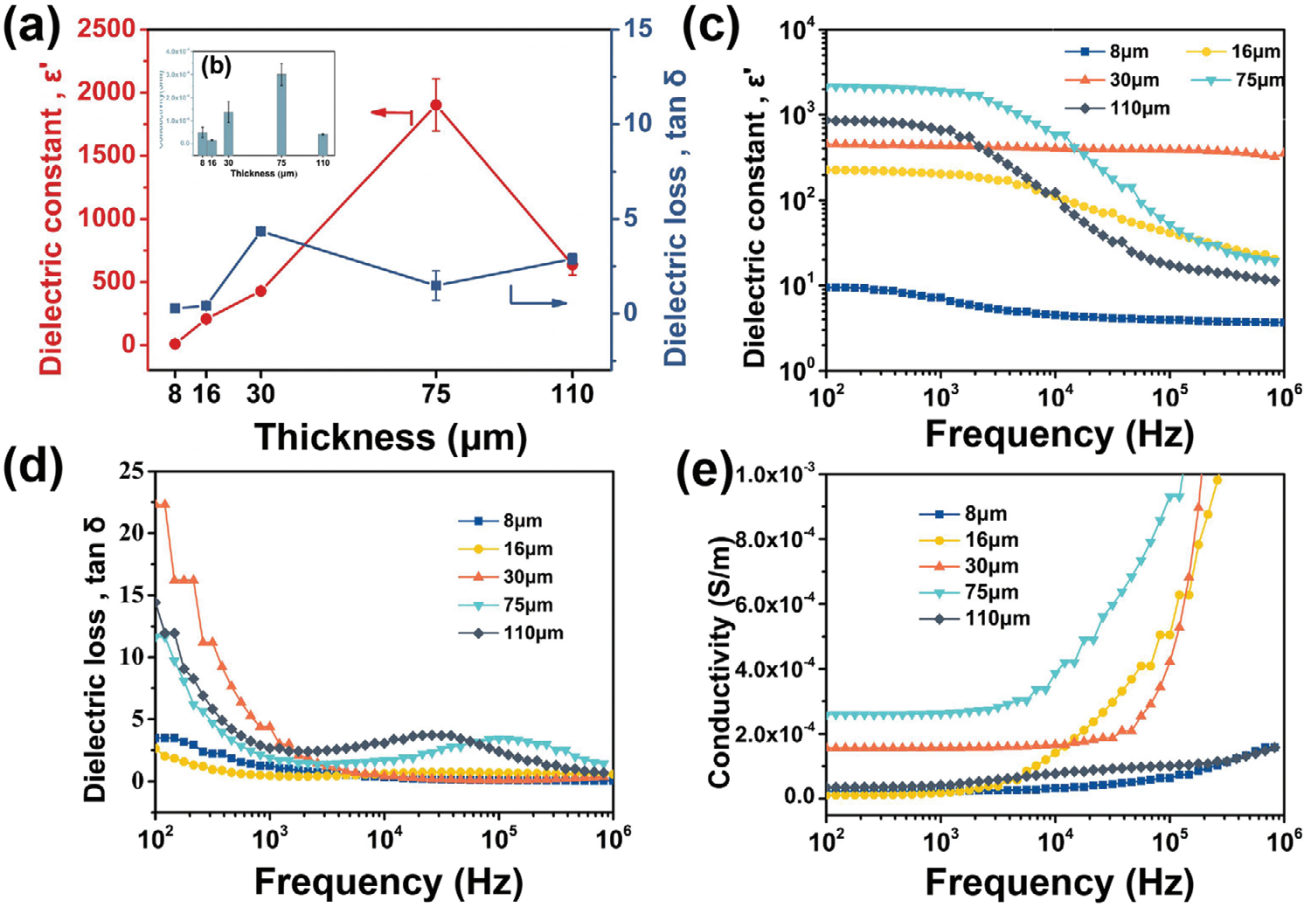
a) The dielectric constant and dielectric loss and b) the conductivity of PANI/PEEK films with different thicknesses from 8 to 110 µm at 1000 Hz and room temperature. Dependence of c) the dielectric constant, d) dielectric loss, and e) conductivity on the frequency of PANI/PEEK films with different thicknesses from 8 to 110 µm at 1000 Hz and room temperature.

After exploring the effects of different film‐forming conditions on the film performance, the best experimental conditions were used on the samples with parallel plate capacitor configuration to further explore the influence of PANI on the thickness of the template during the in‐situ confined growth process. It can be seen from **Figure** **4**a that the dielectric constant first increases and then decreases with the increase of the film thickness at room temperature and 1000 Hz. The dielectric constant of the 75 µm‐PEEK/PANI film reaches the maximum, and the dielectric loss of the 16 µm‐PEEK/PANI film reaches the minimum. In the process of PANI formation, Schiff alkali undergoes hydrolysis, ketimine is converted into ketone and aniline is formed at the same time, and aniline diffuses in the film as the reaction proceeds. When the film is too thin, due to the diffusion of aniline outside the film during the reaction process, part of the aniline cannot form self‐polymerization in the film. There are fewer conductive fillers, and the distance between adjacent conductive particles is larger, which effectively prevents the accumulation of a large number of electrons. When the film becomes thinning (8 µm), holes will be formed in the film, which results in defects in the formed parallel plate capacitor and higher dielectric loss. As the thickness of the film increases, the outward diffusion of aniline is hindered. (Figures S19–S21, Supporting Information) With the thickness of the film increasing to 16 µm, when the pore structure and the content of PANI are balanced, PANI is uniformly dispersed, which forms a film with lower dielectric loss and higher dielectric constant at 16 µm‐PANI/PEEK. With the thickness further increase (30 µm), the PANI content increases, which will cause leakage current, and both the dielectric constant and dielectric loss will increase.^[^ ^38^, ^46^ ^]^ Interestingly, when the film thickness increases to 75 µm‐PANI/PEEK, the film gradually forms a conductive path, the PANI is evenly dispersed, forming a higher dielectric constant and lower dielectric loss. However, when the thickness of the film is further increased more than 110 µm, due to the hindered diffusion of aniline, part of the PANI may react on both sides of the film, which may cause partial agglomeration and affect the formation of conductive paths, thus reducing the permittivity and conductivity. TEM showed the morphology and dispersion of PANI in the film. The PEEKt film was subjected to TEM test by freezing section, and it was found that there was no particulate matter inside the film, and it was relatively uniform. The particles in the 16 µm‐PANI/PEEK film were evenly dispersed. As the thickness increases, the black particles in the 75 µm‐PANI/PEEK film increase, and the PANI gradually increases. When the thickness is further increased, 120 µm‐PANI/PEEK forms fibrous PANI with less content, which further verifies the SEM characterization. It can be known that a thinner film can make the resistance of molecules entering the film smaller, and the resulting PANI/PEEK composite film has better dispersibility. When the thickness reaches a certain level, the PANI increases and gradually forms a pathway. The dielectric and conductivity test of the film has a significant impact. The 16 µm‐PANI/PEEK film has better dielectric properties, with the dielectric constant of 205.4 and the dielectric loss of 0.401. As shown in Figure 4c,d, the K value of all composites decreases with the increase of AC frequency.^[^ ^38^, ^39^, ^47^ ^]^ As shown in Figure 4d, the D value of the composites shows a trend of decreasing with the increase of AC frequency, and a phenomenon of local increase occurs at high frequency. When the film thickness increases and conductive fillers gradually increase, the dielectric material will be polarized under the action of an external electric field, which is caused by the directional distribution of certain dipoles.^[^ ^46^ ^]^ However, as the frequency increases, the electric field changes rapidly and the period is very short, which makes the dipole's steering movement unable to keep up with the change of the electric field due to internal resistance.^[^ ^68^ ^]^ At the same time, due to the increase in thickness, PANI forms a conductive path. When the thickness increases more than 75 µm, a leakage current phenomenon occurs due to the formation of a conductive path. The dielectric loss forms a loss peak at high frequencies. This phenomenon may cause by the interface polarity, which is related to the relaxation effect.^[^ ^39^, ^47^ ^]^

Figure 4b shows the relationship between electrical conductivity and PEEKt film thickness at room temperature and 1000 Hz, which shows the conductivity of the 75 µm‐PEEK/PANI film reaching the maximum.^[^ ^69^, ^70^, ^71^ ^]^ As the thickness increases to 75 µm, the aniline on the outside of the film diffuses into the solution, but the aniline on the inside is restricted to the inside of the film due to diffusion hindered, and the formed PANI gradually aggregates into a conductive path, and the conductivity increases. The conductivity of the 75 µm‐PANI/PEEK film reaches a maximum of 3.01×10^−4^ S m^−1^. When the thickness increases again more than 110 µm, less PANI is formed in the film and the conductivity decreases, which is because the diffusion of oxidant molecules and aniline molecules is hindered. It can be clearly seen from Figure 4e that the conductivity values of the films of all thicknesses increase with the increase of the AC frequency, and the conductivity remains unchanged at low frequencies. This shows that the prepared PANI/PEEK materials have significant applications as electronic packaging materials and embedded capacitors.

### 2.5 Potential Application of PANI/PEEK Film in Industrial Production

To further understand the mechanical properties of composite materials, we conducted the tensile strength test (Table S3, Supporting Information). The tensile strengths of 16 µm‐PANI/PEEK and 75 µm‐PANI/PEEK composite films are 75.8 and 75.0 MPa, respectively, which are equivalent to pure PEEK commercial films, and even 70.8 MPa for commercial PEEK films. However, due to the presence of pores in the composite film, the elongation at the break of the PANI/PEEK film is much lower than that of the PEEK film. The base material of the PANI/PEEK composite film is PEEK, and its excellent mechanical properties are an important reason for the high tensile strength of the film. At the same time, the amino group of PANI and the ketone group of PEEK will form hydrogen bonds and interact at the interface to increase the rigidity of the composite film. Compared with other high‐dielectric composite films, the tensile strength is maintained at a higher level.

The content of PANI is increased by the method of confined polymerization. We used PANI/PEEK powder to prepare the PANI/PEEK composite board material by hot pressing technology (Figure S22, Supporting Information). This shows that the use of this method can further realize industrialized mass production and has a wide range of application prospects for dielectric, conductive materials, and electronic packaging materials. At the same time, this work provides a method for one‐step conversion of materials containing ketimine structure to PANI, which further broadens the application prospects of materials.

## 3 Conclusion

Overall, based on confined polymerization, we have designed the PANI/PEEK film through one‐step method and in situ growth. The hydrolysis is used to produce aniline monomer inside the template of PEEKt film, which realizes the in situ and restricted growth and polymerization of PANI inside the film. The problem that PANI maintains a high content in the film while achieving uniform dispersion and not easy to agglomerate is realized. This work further explores the growth and aggregation mode of PANI in the process of domain‐limited aggregation. Through HRTEM, DMA, and XRD, the micro‐nano structure changes during the growth of PANI were further analyzed. Through analysis, we found that during the co‐growth of PANI and PEEK, PANI grew in the free volume of PEEK, effectively restricting the movement of the PEEK chain. On the basis of the best experimental conditions, the influence of different thicknesses on the growth of PANI film was adjusted, and the dielectric constant of 16 µm‐PANI/PEEK was 205.4 (dielectric loss 0.401), and the conductivity of 75 µm‐PANI/PEEK reached 3.01×10^−4^ S m^−1^. In addition, taking full advantage of the PEEK substrate, the tensile strength of the PANI/PEEK film reached 75.8 MPa. This kind of material has potential application value in the application of high dielectric materials and conductive materials.

## 4 Experimental Section

Detailed experimental information is reflected in the supporting information.

## Conflict of Interest

The authors declare no conflict of interest.

## Supporting information

Supporting Information

## Data Availability

Research data are not shared.
