# Supplementary material for: Based On Confined Polymerization: In Situ Synthesis of PANI/PEEK Composite Film in One‐Step
Source: Adv Sci (Weinh). 2021 Nov 11;9(1):2103706. doi: 10.1002/advs.202103706 (PMC8728828; doi:10.1002/advs.202103706)
Supplement: Supplementary file 1 — Supporting Information [file ADVS-9-2103706-s001.pdf]

ADVANCED SCIENCE
Open Access

Supporting Information

for *Adv. Sci.*, DOI: 10.1002/advs.202103706

Based on confined polymerization: In-situ synthesis of PANI/PEEK composite film in one-step

Ziyu Lin, Ning Cao, Zhonghui Sun, Wenying Li, Yirong Sun, Haibo Zhang, Jinhui Pang and Zhenhua Jiang*

Supporting Information

Based on confined polymerization: In-situ synthesis of PANI/PEEK composite film in one-step

Ziyu Lin, Ning Cao, Zhonghui Sun, Wenying Li, Yirong Sun, Haibo Zhang, Jinhui Pang* and Zhenhua Jiang

((Please insert your Supporting Information text/figures here. Please note: Supporting Display items, should be referred to as Figure S1, Equation S2, etc., in the main text…)

Experimental Procedures

1. Chemicals and Materials

Nmethyl-2-pyrrolidinone (NMP), ethanol (EtOH), methanol (MeOH) hydrochloric acid (HCl) and potassium carbonate (K_2CO_3) were purchased from Sinopharm Chemical Reagent Co., Ltd. (China). Ammonium persulfate ($(NH_4)_2S_2O_8$, APS), sulfolane, aniline, hydroquinone and 4,4'-Difluorobenzophenone were purchased from Aladdin. All reagents and solvents are commercially available and were used without further purification before use.

2. Characterization methods

2.1 Nuclear Magnetic Resonance Spectrometer (NMR)

The purity and structure of monomers and polymers were tested by nuclear magnetic resonance spectroscopy. The monomers and polymers were dissolved in deuterated DMSO and passed through a nuclear magnetic resonance spectrometer (model Avance NEO, purchased from Swiss BRUKER Instrument Company, and the test condition was 400 Hz)

2.2 Fourier transform infrared spectrometer (FTIR)

The functional groups of all materials are tested by a Fourier transform infrared spectrometer, the instrument model is iS10, purchased from Thermo Fisher Scientific. The test mold is in total reflection mode.

2.3 Nano infrared system (Nano-IR)

The nano-infrared spectrum and AFM phase diagram of the PANI/PEEK film section were tested by nano-infrared system, the instrument model is NanoIR3, purchased from Bruker. The section of the PANI/PEEK membrane was sliced by cryostat under normal temperature mode, and the slice thickness was 500 nm.

2.4 Mass loss analysis

The content of PANI in the PANI/PEEK film was calculated by weight loss. The film before and after the treatment was weighed by an electronic balance and the PANI content was calculated. Three parallel measurements for each film.

2.5 Wide-angle X-ray diffractometer (WXRD)

Wide-angle X-ray diffractometer (WXRD) were analyzed by Empyrean purchased from PANalytical B.V. The 2θ scan data were collected at 6 °/min intervals over ranges of 10 °–50 °. XRD calculation layer spacing is calculated by Formula (S1)[S1]:

$$2d\sin\theta=n\lambda$$

d: distance between crystal planes; θ: read out on the picture; x-ray wavelength n=1; λ

=0.15406 nm

2.6 X-ray photoelectron spectra (XPS)

The surface structure of PANI/PEEK film was analyzed by XPS. X-ray photoelectron (XPS) spectra were collected using a Thermo ESCALAB 250 equipped with an Al Ka X-ray source (1486.6 eV).

2.7 Dynamic Mechanical Analyzer (DMA)

The Tg of the film was tested by DMA, and the instrument model was RSAG2, which was purchased from TA Company in the United States. The film was cut into a 5 mm*40 mm sample, the test temperature was 50 °C-300 °C, the test condition was tensile mode, and the strain was 0.005 %.

2.8 Transmission electron microscope (TEM)

The internal structure of PANI/PEEK films of different thicknesses was tested by TEM. The instrument model was TECNAIG2 F20 S-TWIN, purchased from FEI ELECTRON OPTICS in the Netherlands. The film was sectioned by a cryostat before the test, the sectioning mode was frozen (-100 °C) section, and the thickness was 50 nm.

2.9 High resolution transmission electron microscope (HRTEM)

The lattice structure of the PANI/PEEK film was tested by HR-TEM, the instrument model was FEI Tecnai G2 F20, purchased from FEI Company in the United States. The film was sectioned by a cryostat before the test, the sectioning mode was frozen (-100 °C) section, and the thickness was 30 nm.

2.10 Selected area electron diffraction (SAED)

The structure of the PANI/PEEK film was tested by electron diffraction, and the instrument model was FEI Tecnai G2 F20, which was purchased from FEI Company in the United States. The film was sectioned by a cryostat before the test, the sectioning mode was frozen (-100 °C) section, and the thickness was 30 nm.

2.11 Scanning electron microscope (SEM)

The surface morphology of the sample was observed by the scanning electron microscope (SEM/FEI company, USA, Model Nova nano 450). Before testing, the sample was sprayed with Platinum for 60 seconds. The cross section was that the films were quenched in liquid nitrogen.

2.12 Dielectric and conductivity testing

Dielectric and conductive properties were measured using an Agilent LCR meter (4294A) and the sample films (diameter 7.0 mm) were deposited on 500 Å thick copper metal. Each sample was tested 4 times in parallel.

2.13 Density test (ρ)

The density of the film was tested by a densitometer, the model is SD-200L-solid density meter, purchased from Japan's ALFA MIRAGE company. The density of the film was tested by buoyancy.

2.14 Tensile strength test

The rectangle sample of the 5 mm (width) *25 mm (length) was cut from the PANI/PEEK film to test the tensile strength using the electronic universal testing machine (TA company,

USA, Model RSA-G2). Each sample was tested 4 times in parallel. The gap distance of the sample was 8 mm, and the tensile speed was 2 mm/min at room temperature.

2.15 Frozen Ultrathin Microtome

The film was sliced to prepare a test sample. The film was wrapped in epoxy resin, and sliced with a Leica cryo-microtome, the instrument model was EM FC7, which was purchased from Leica Company.

2.16 Elemental analysis (CHN)

The elemental analysis (CHN) of the film was tested by the combustion method (Model Vario EL cube/ Elementar company). The O element is calculated from the total content.

2.17 N_2 Adsorption test

The 100mg sample was degassed at 150 ^{o}C for 12 hours, and then tested at 77K by N2 adsorption equipment which is the Autosorb-iQ3 (Quantachrome Instruments).

3. Synthesis

3.1 Synthesis of N-phenyl (4,4′-difluorodiphenyl) ketamine

According to previous research [S2], N-phenyl (4,4′-difluorodiphenyl) ketamine was successfully synrhesis. For specific synthetic steps, the **Scheme S1** could be found. 21.82 g (0.10 mol) of DFBP, 13.7 mL (0.15 mol) of aniline, 50.00 g of molecular sieves (4 Å), and 80 mL of toluene were added into a 250 mL three-neck round-bottomed flask fitted with an argon inlet/outlet, a Dean-Stark trap, a mechanical stirrer and a reflux condenser. Then the reaction temperature was heated at 160 °C for 24 h, and fell to room temperature (RT). After filtrating to remove the molecular sieves, the mixture was evaporated by rotary evaporation. Then the crude product was recrystallized by methanol for three times. The yellow product was obtained by drying in vacuum oven at 60 °C for 12 h. (23.76 g, 0.08 mol). Yield: 81 %.

3.2 Synthesis of Poly (aromatic ether amine) (PEEKt)

Through polymerization, poly (aromatic ether amine) (PEEKt) was successfully prepared. More details of synthesis steps could be found from the **Scheme S2**. [S3.S4] 2.2022 g (20 mmol) of the 1,4-benzenediol monomer, 5.2796 g (20 mmol) of N-Phenyl (4,4′-difluorodiphenyl) ketamine, 3.3168 g of K_2CO_3, 12.0 mL of toluene, and 19.8 mL of sulfolane were charged in a 100 ml three-necked flask equipped with mechanical stirring, a Dean-Stark trap and a reflux condenser. Under nitrogen atmosphere, the reaction was heated to 155 °C to carry water for 3 hours to ensure that the water in the reaction was completely removed. The reaction was warmed to 210 °C for 7-9 h, and waited until the reaction system became a dark brown viscous liquid. Finally, the solution in the reaction was poured into deionized water to obtain a pale green flocculent solid. The strip-shaped solid was pulverized, and then washed by deionized water 5 times at room temperature conditions for 60 minutes each time. The

product was placed in a vacuum oven at 60 °C for 24 h to completely remove water. A yellowish solid was finally obtained. The polymerization yield was as high as 97 %.

3.3 Preparation of PEEKt films

The films preparation and oxidation process were shown in **Figure 1** and **Scheme S3**. The film was prepared with solution casting method. PEEKt was dissolved in DMAC solution (15 mL) and stirred at room temperature until completely dissolved. The solution was allowed to stand at room temperature for 12 h to ensure that bubbles were completely released. The solution was poured on a clean glass plate (10 cm*10 cm) and placed in an oven at 80 ºC. The oven was maintained at 80 ºC for 12 h and 120 ºC for 24 h, and then cooled to room temperature. The film was placed in deionized water coagulation bath until it was detached naturally. Finally, film with the different thickness was obtained.

3.4 Preparation of PANI/PEEK films

The obtained PEEKt films were subjected to oxidation treatment to prepare PANI / PEEK composite films in one step. The weighed $(NH_4)_2S_2O_8$ solid was dissolved in HCl and stirred until the solid was completely dissolved. The PEEKt film obtained before was cut into a size of 2 cm*2 cm and placed in a 25 ml airtight glass bottle, and then 20 ml of the prepared solution was added to it. The closed sample bottle was left in the oven for a certain period of time, and then cooled to room temperature. A detailed description of films manufacturing conditions was shown in the Supporting Information (**Table S4-S7**). Under the ultrasonic cleaner, the obtained composite films were washed with water and ethanol 3-5 times, each time for 15 minutes. Finally, the films were dried in 80 ºC vacuum oven for later use.

3.5 Preparation of 220- PANI/PEEK films

PANI/PEEK films prepared previously were annealed. The previously prepared 75 μm-PANI/PEEK film was placed in a high temperature oven at 220 °C and treated with high temperature vacuum for 5 hours. After cooling slowly, 220-PANI/PEEK film was obtained.

3.6 Preparation of PEEKt(HCl) films

The previously prepared PEEKt film was cut into a size of 2 cm*2 cm, and placed in 20 ml, 2.5 M HCl solution, and reacted at 60 °C for 6 hours. Take it out and ultrasonically clean it with ethanol and water 3 times.

3.7 Statistics Analysis

Each experiment was tested three times in parallel. All experimental results requiring quantitative analysis were analyzed using statistical product and service solution 13.0 (SPSS 13.0) software. Data were expressed as mean ± standard error of the mean.

4 Supporting Schemes

Scheme S1. Synthesis of N-phenyl (4,4′-difluorodiphenyl) ketimine

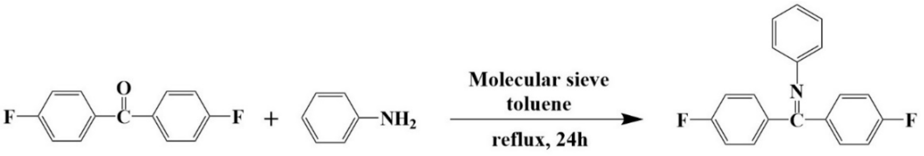

Scheme S2. Synthesis of intermediate polymer PEEKt

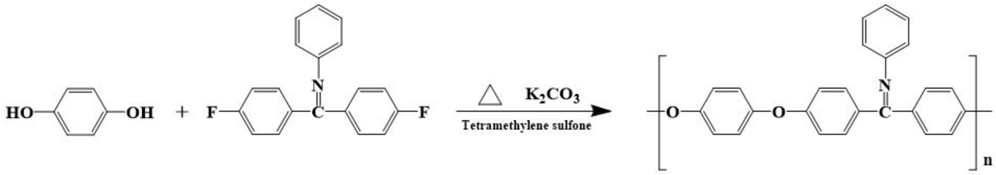

Scheme S3. PEEKt films oxidation to synthesize PANI / PEEK composite films

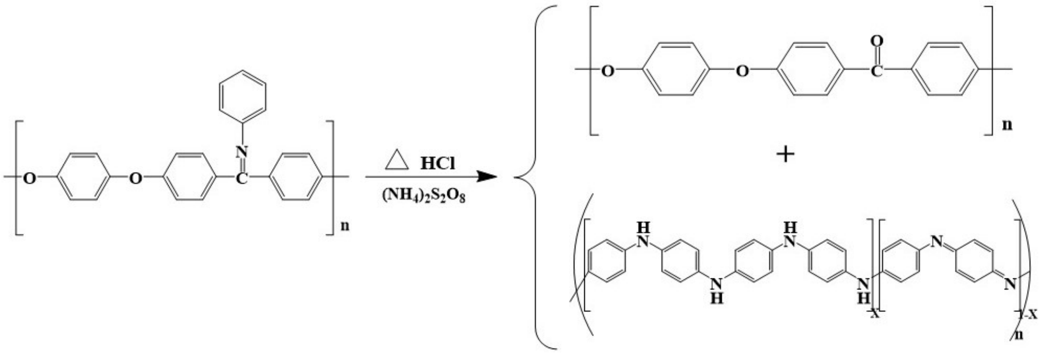

5 Supporting Figures

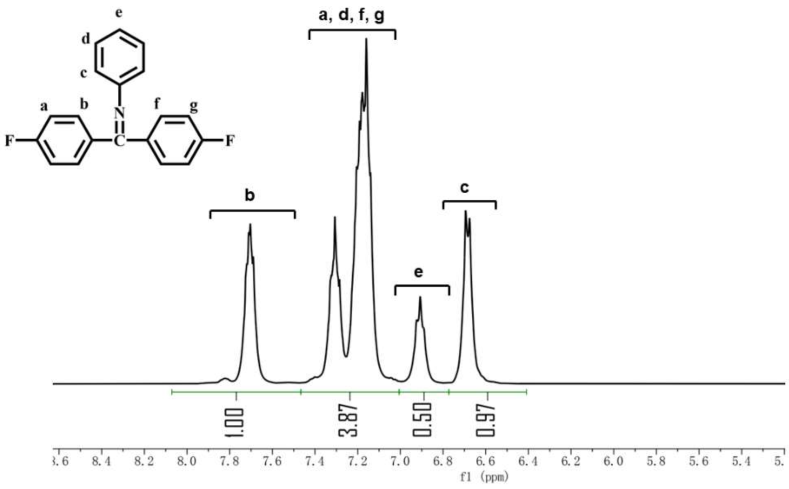

Figure S1. ^{1}H NMR spectrum of N-phenyl(4,4-difluorodiphenyl) ketimine.

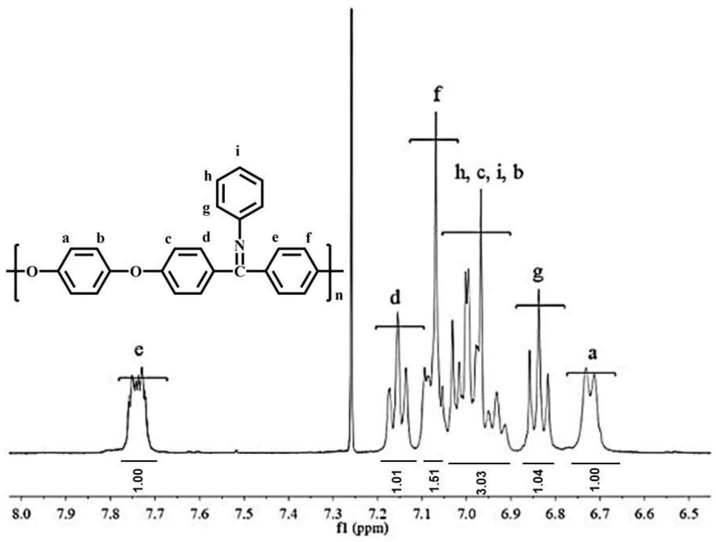

Figure S2. ^{1}H NMR spectrum of PEEKt.

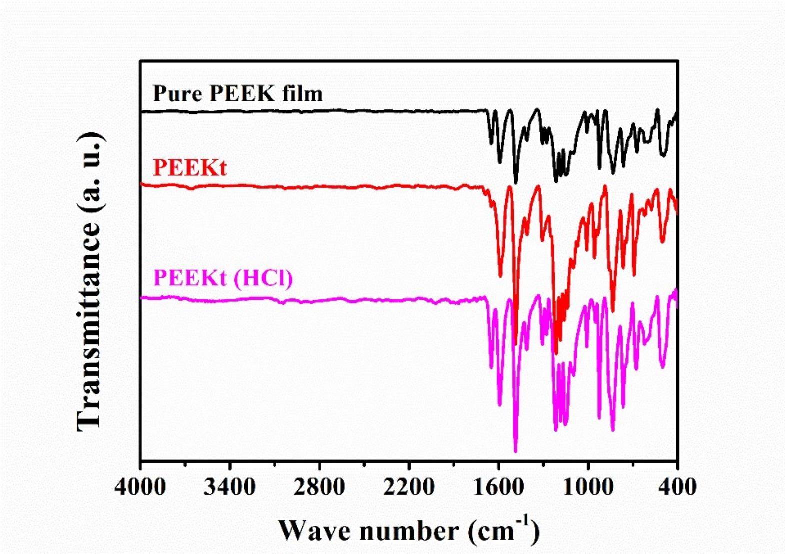

Figure S3. ATR-FTIR spectrum of pure PEEK film, PEEKt film, and PEEKt film treated with hydrochloric.

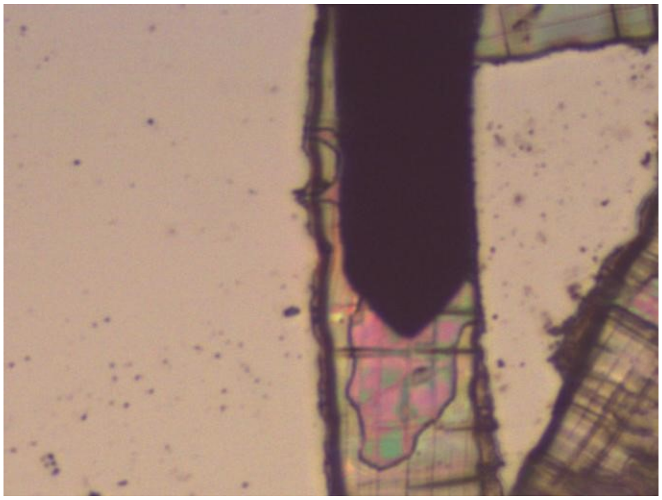

Figure S4. Picture nano-IR of the cross-sectional of 75 μm-PANI/PEEK film.

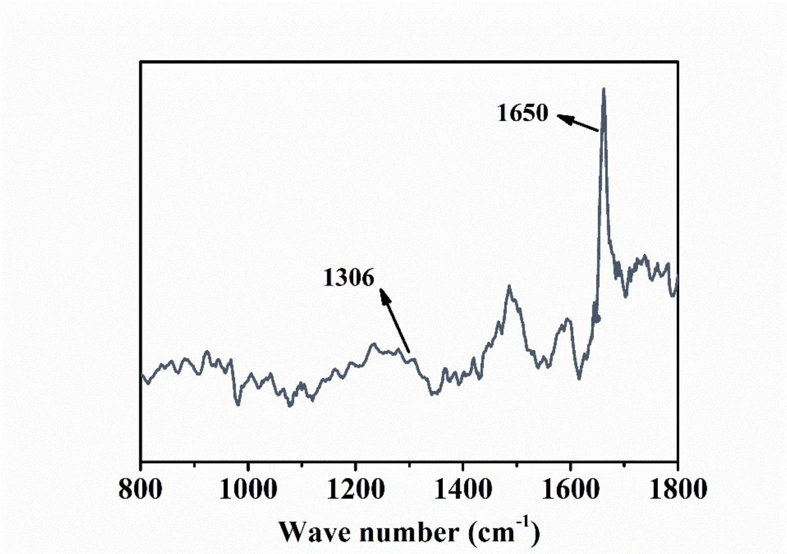

Figure S5. Nano-IR spectrum of the cross-sectional of 75 μm-PANI/PEEK film.

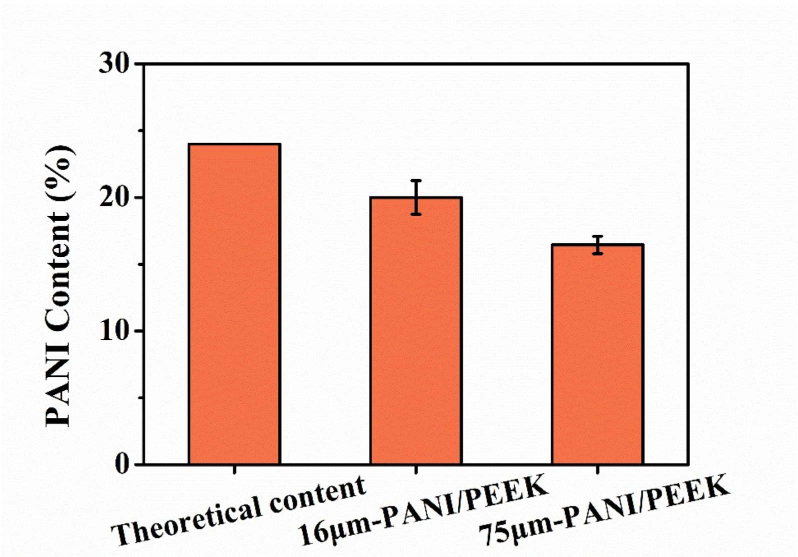

Figure S6. Mass loss analysis. The PANI content of PANI/PEEK film.

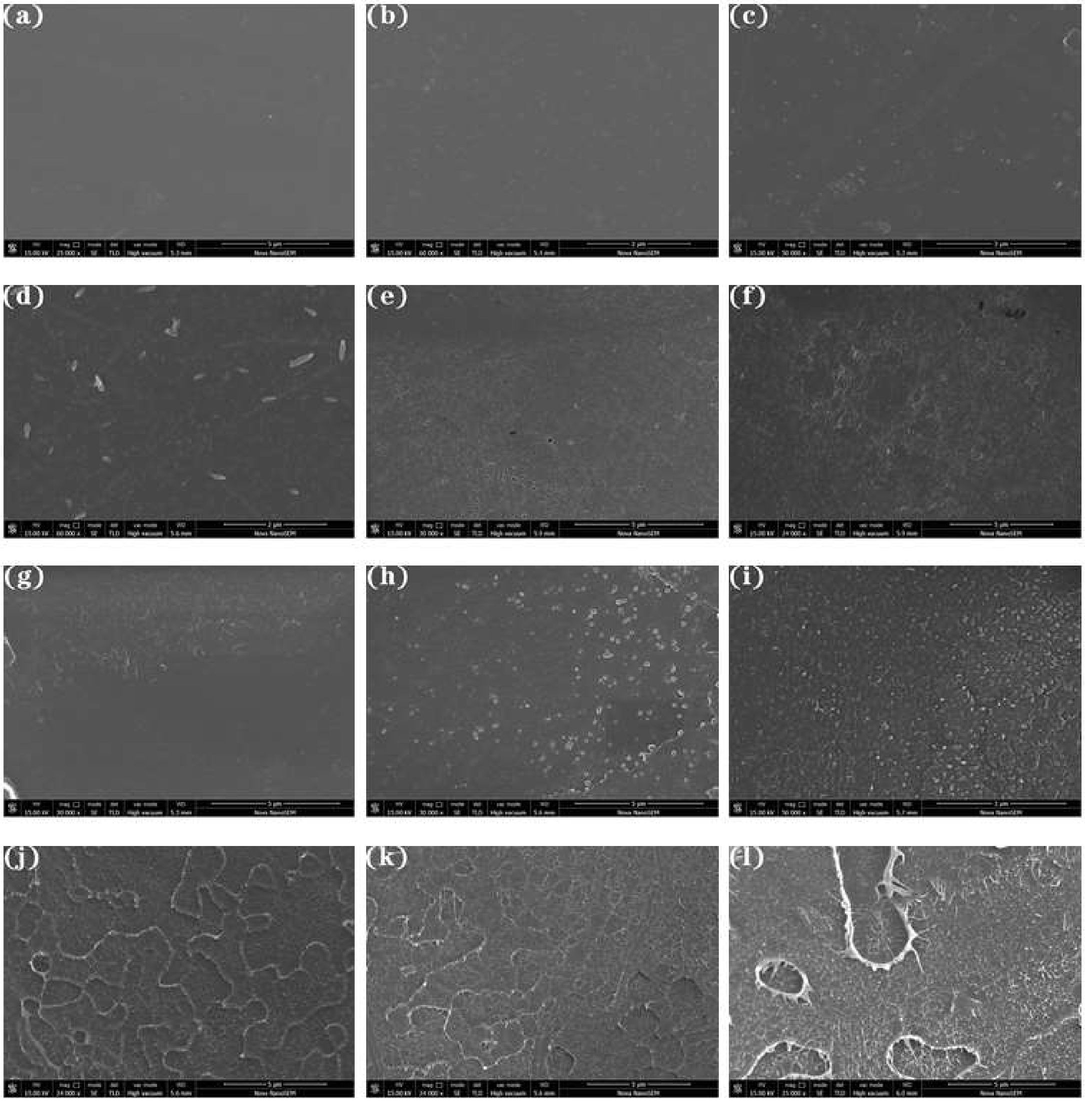

Figure S7. SEM of the as-prepared films: PANI/PEEK films with different treatment time. (a) 10 min, (b) 30 min, (c) 60 min, (d) 240 min, (e) 360 min, (f) 480 min were the top surface SEM images of films. Scale bar was 5 μm; (g) 10 min, (h) 30 min, (i) 60 min, (j) 240 min, (k) 360 min, (l) 480 min were the cross-sectional SEM images of films. Scale bar was 5 μm.

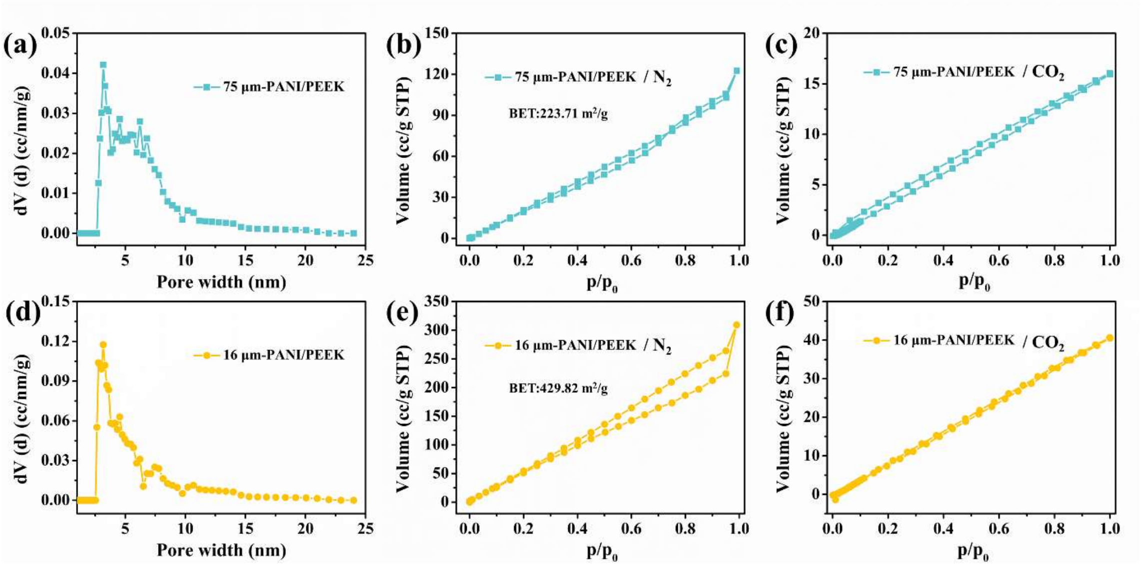

Figure S8. (a) Pore size distribution analysis (b) N_2 adsorption–desorption isotherms (c) CO_2 adsorption–desorption isotherms for 75 μm-PANI/PEEK; (d) Pore size distribution analysis (e) N_2 adsorption–desorption isotherms (f) CO_2 adsorption–desorption isotherms for 16 μm-PANI/PEEK.

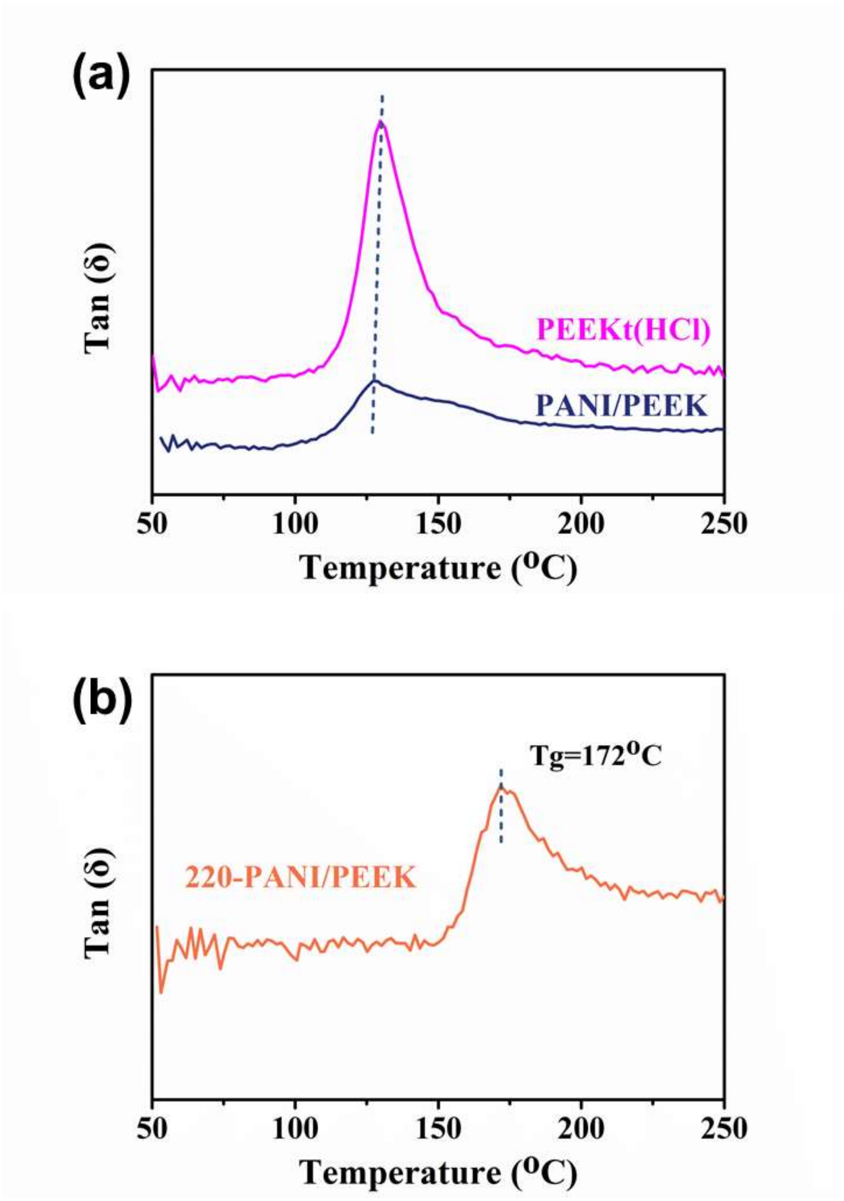

Figure S9. DMA of (a) PEEKt film treated with hydrochloric and PANI/PEEK film. (b) 220-PANI/PEEK (The PANI/PEEK film was annealed at 220 °C).

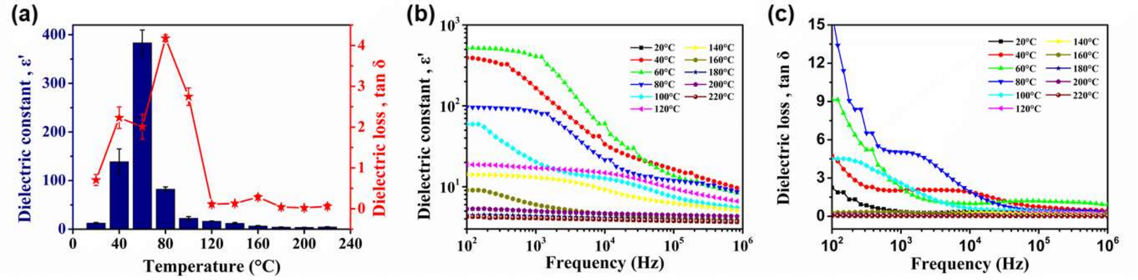

Figure S10. (a)The dielectric constant and dielectric loss of PANI/PEEK films with different treatment temperature from 20 ºC to 220 ºC at 1000 Hz and room temperature. Dependence of (b) the dielectric constant and (c) dielectric loss on the frequency of PANI/PEEK films with different treatment temperature from 20 ºC to 220 ºC at 1000 Hz and room temperature.

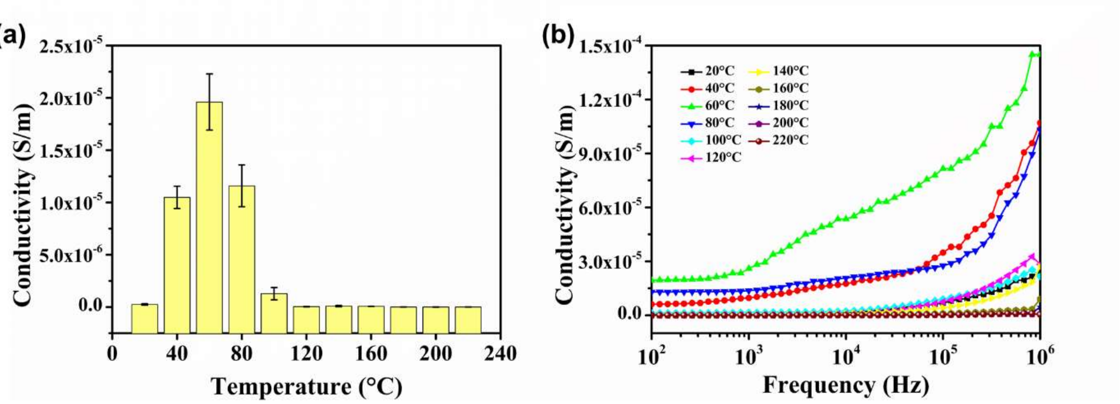

Figure S11. (a) The conductivity of PANI/PEEK films with different treatment temperature from 20 ºC to 220 ºC at 1000 Hz and room temperature. (b)Dependence of conductivity on the frequency of PANI/PEEK films with different treatment temperature from 20 ºC to 220 ºC at 1000 Hz and room temperature.

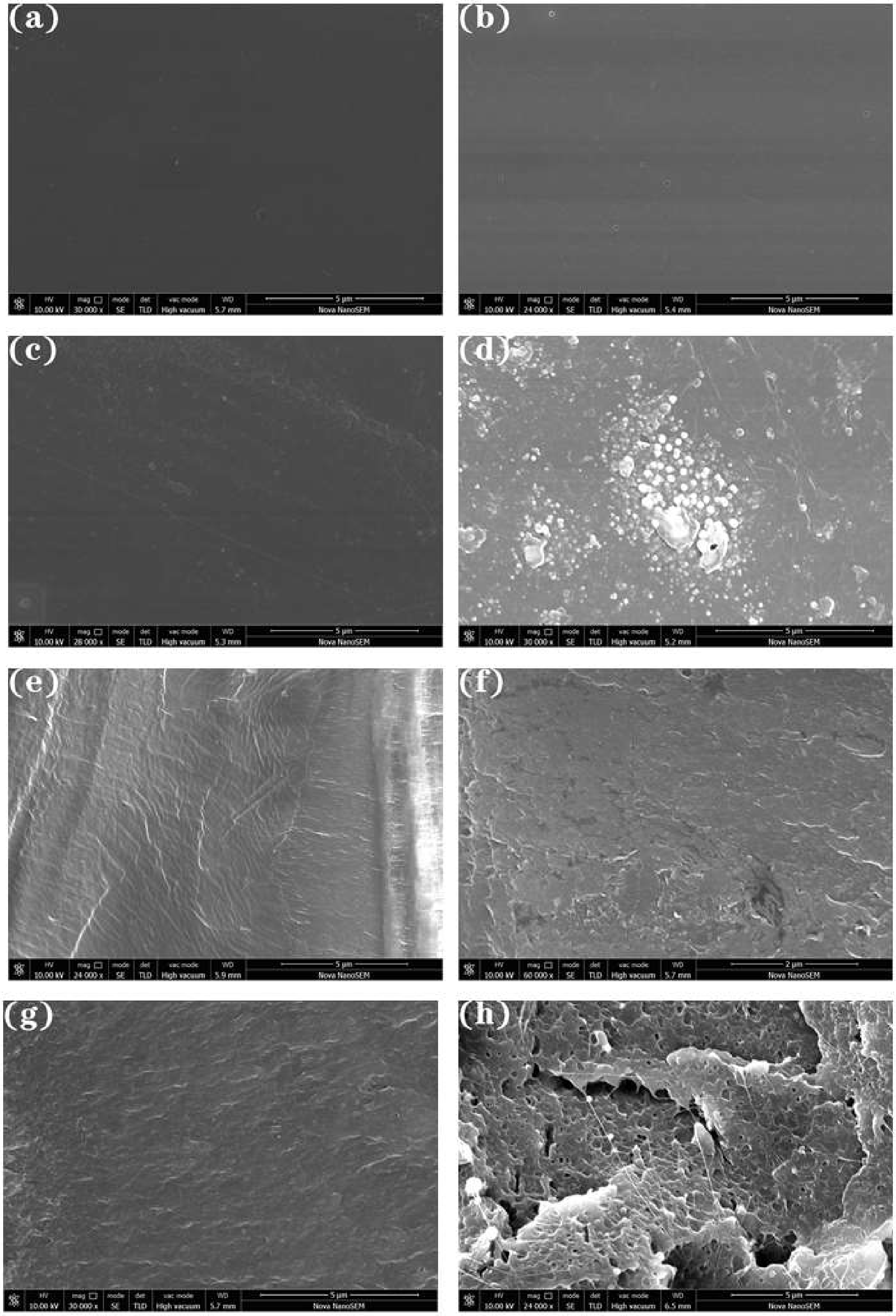

Figure S12. SEM of the as-prepared films: PANI/PEEK films with different treatment temperature. (a) PEEKt, (b) 20 ºC, (c) 60 ºC, (d) 140 ºC were the top surface SEM images of films. Scale bar was 5 μm; (e) PEEKt, (f) 20 ºC, (g) 60 ºC, (h) 140 ºC were the cross-sectional SEM images of films. Scale bar was 5 μm.

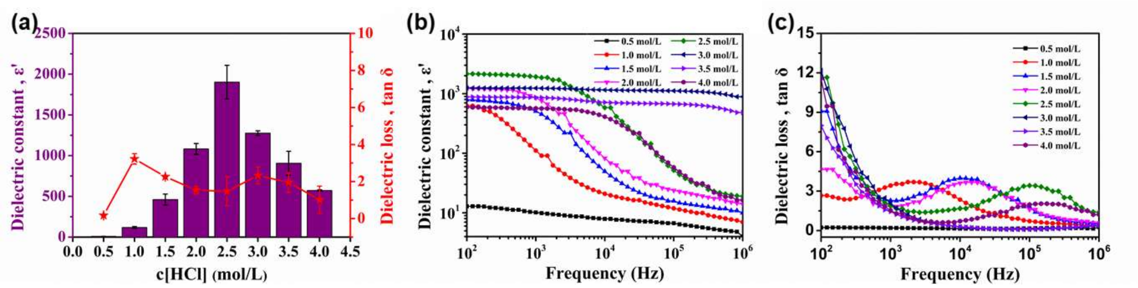

Figure S13. (a)The dielectric constant and dielectric loss of PANI/PEEK films with different treatment c(HCl) from 0.5 mol/L to 4.0 mol/L at 1000 Hz and room temperature. Dependence of (b) the dielectric constant and (c) dielectric on the frequency of PANI/PEEK films with different treatment c(HCl) from 0.5 mol/L to 4.0 mol/L at 1000 Hz and room temperature.

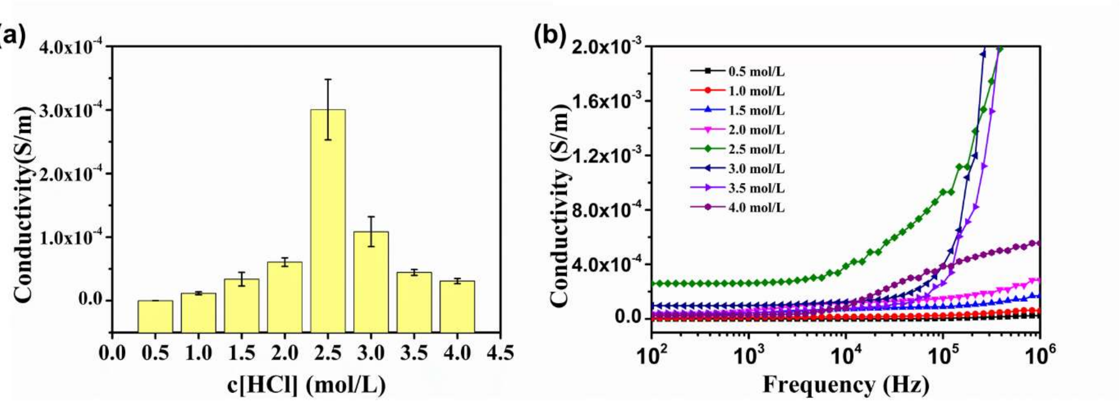

Figure S14. (a) The conductivity of PANI/PEEK films with different treatment c(HCl) from 0.5 mol/L to 4.0 mol/L at 1000 Hz and room temperature. (b)Dependence of conductivity on the frequency of PANI/PEEK films with different treatment c(HCl) from 0.5 mol/L to 4.0 mol/L at 1000 Hz and room temperature.

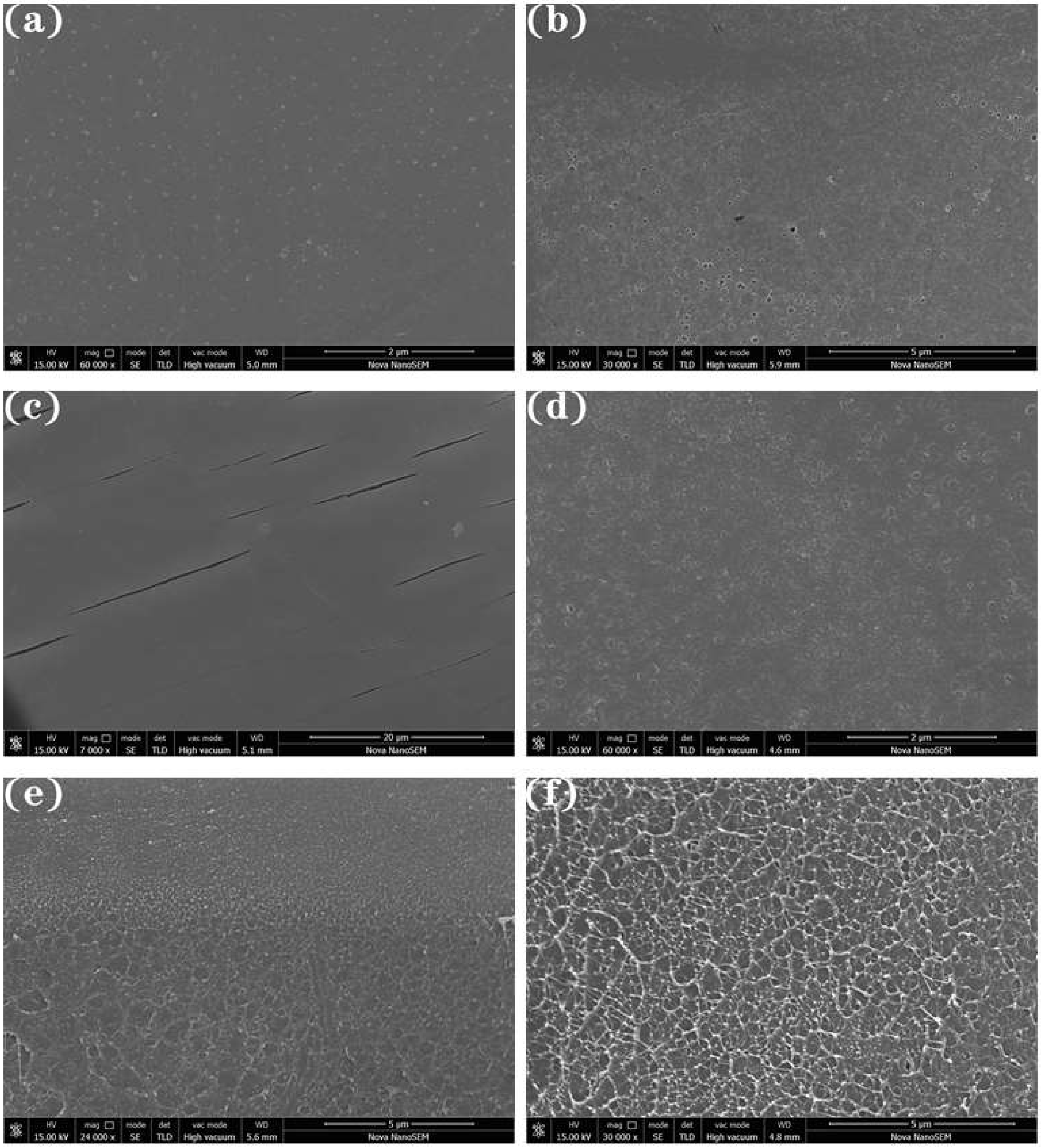

Figure S15. SEM of the as-prepared films: PANI/PEEK films with different treatment c(HCl). (a) 1.0 mol/L, (b) 2.5 mol/L, (c) 4.0 mol/L were the top surface SEM images of films. Scale bar was 5 μm; (d) 1.0 mol/L, (e) 2.5 mol/L, (f) 4.0 mol/L were the cross-sectional SEM images of films. Scale bar was 5 μm.

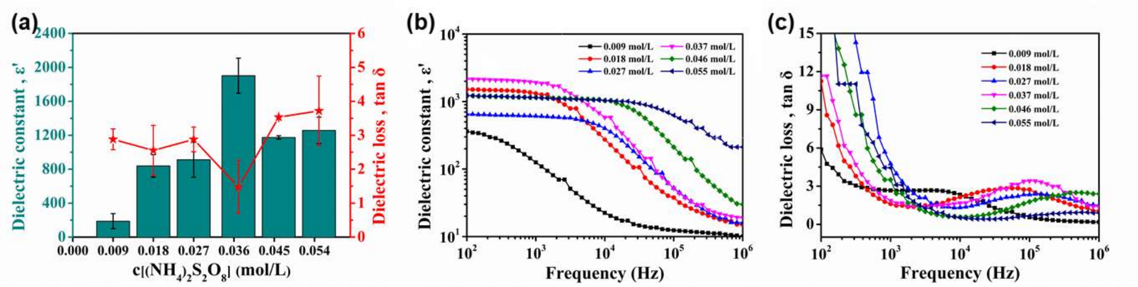

Figure S16. (a)The dielectric constant and dielectric loss of PANI/PEEK films with different treatment c[$(NH_4)_2S_2O_8$] from 0.009 mol/L to 0.055 mol/L at 1000 Hz and room temperature. Dependence of (b) the dielectric constant and (c) dielectric loss on the frequency of PANI/PEEK films with different treatment c[$(NH_4)_2S_2O_8$] from 0.009 mol/L to 0.055 mol/L at 1000 Hz and room temperature.

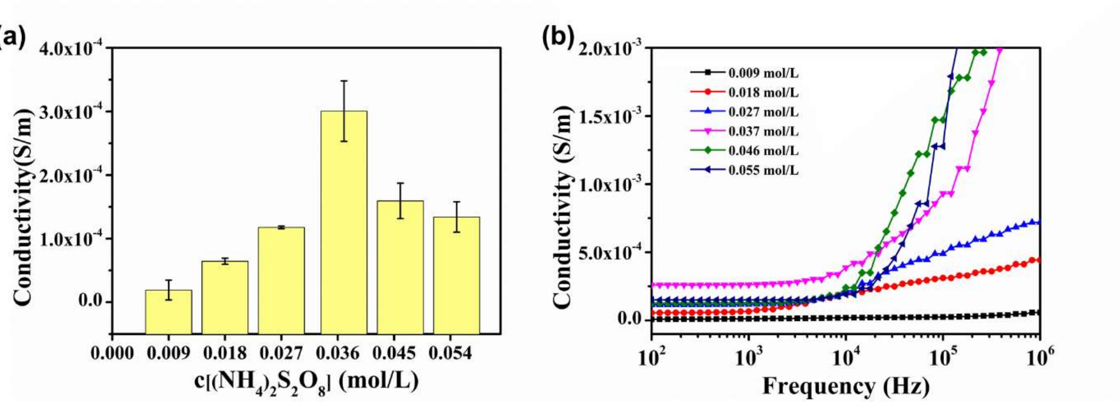

Figure S17. (a) The conductivity of PANI/PEEK films with different treatment c[$(NH_4)_2S_2O_8$] from 0.009 mol/L to 0.063 mol/L at 1000 Hz and room temperature. (b) Dependence of conductivity on the frequency of PANI/PEEK films with different treatment c[$(NH_4)_2S_2O_8$] from 0.009 mol/L to 0.063 mol/L at 1000 Hz and room temperature.

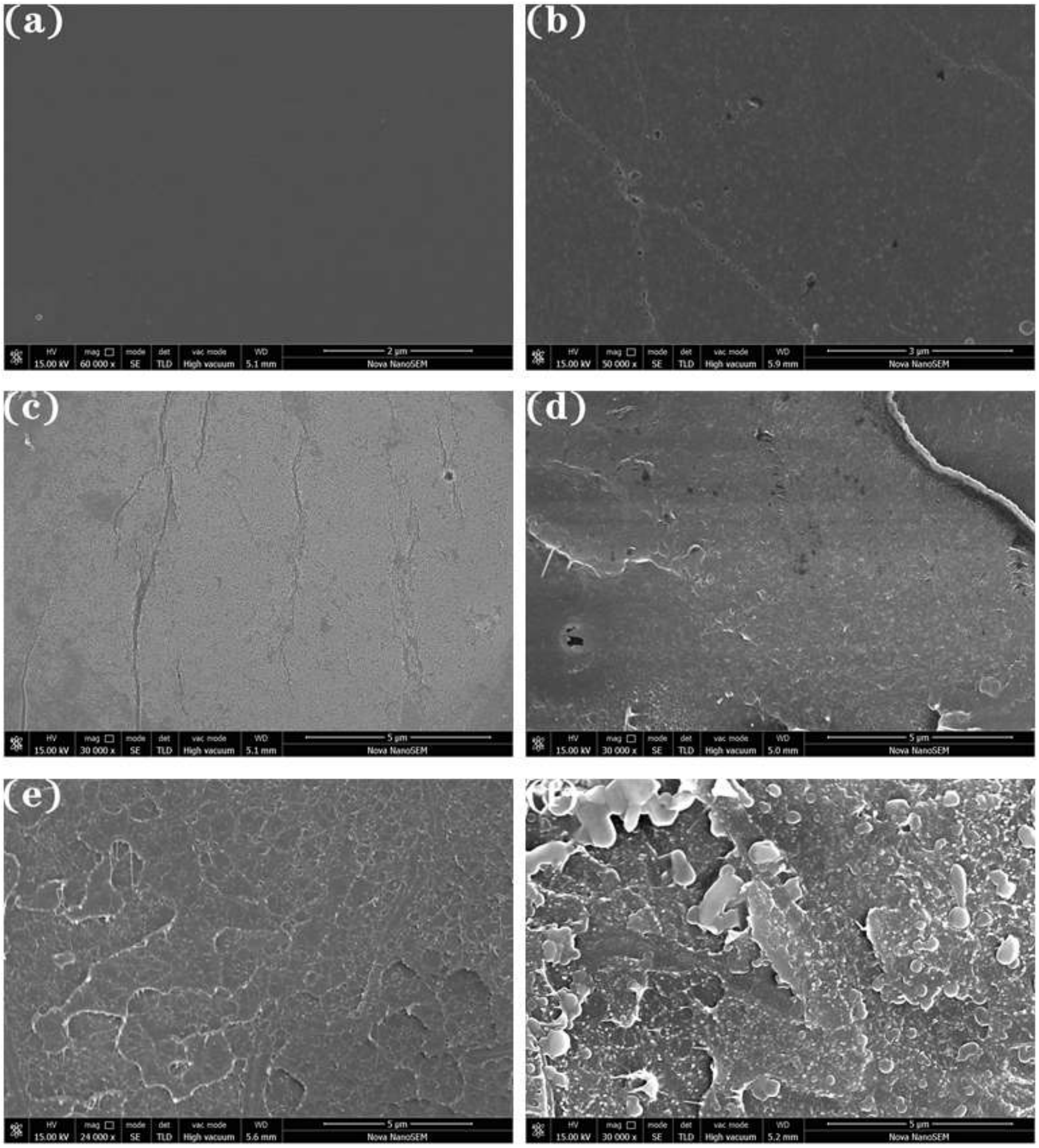

Figure S18. SEM of the as-prepared films: PANI/PEEK films with different treatment c[$(NH_4)_2S_2O_8$]. (a) 0.009 mol/L, (b) 0.037 mol/L, (c) 0.055 mol/L were the top surface SEM images of films. Scale bar was 5 μm; (d) 0.009 mol/L, (e) 0.037 mol/L, (f) 0.055 mol/L were the cross-sectional SEM images of films. Scale bar was 5 μm.

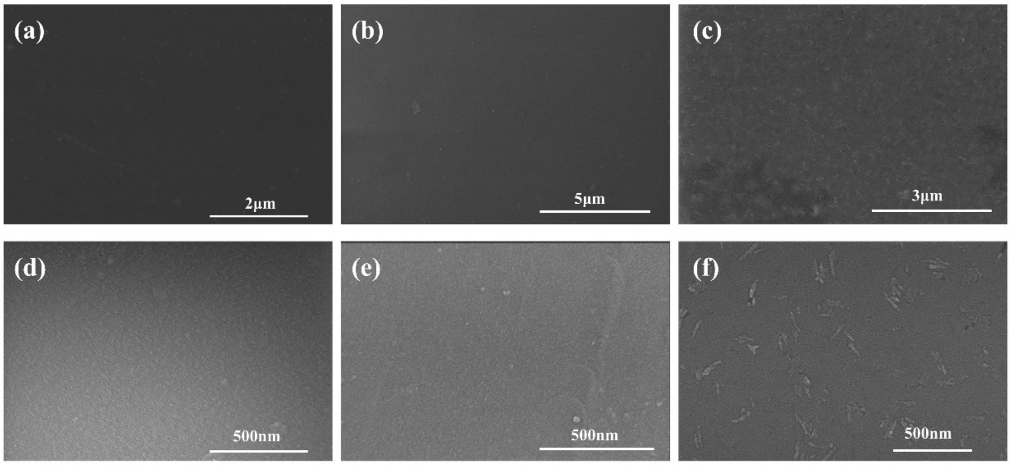

Figure S19. SEM of the as-prepared films: PANI/PEEK films with different thickness. (a) 16μm, (b) 75 μm, (c) 110 μm, (d) 16μm, (e) 75 μm, (f) 110 μm were the top surface SEM images of films.

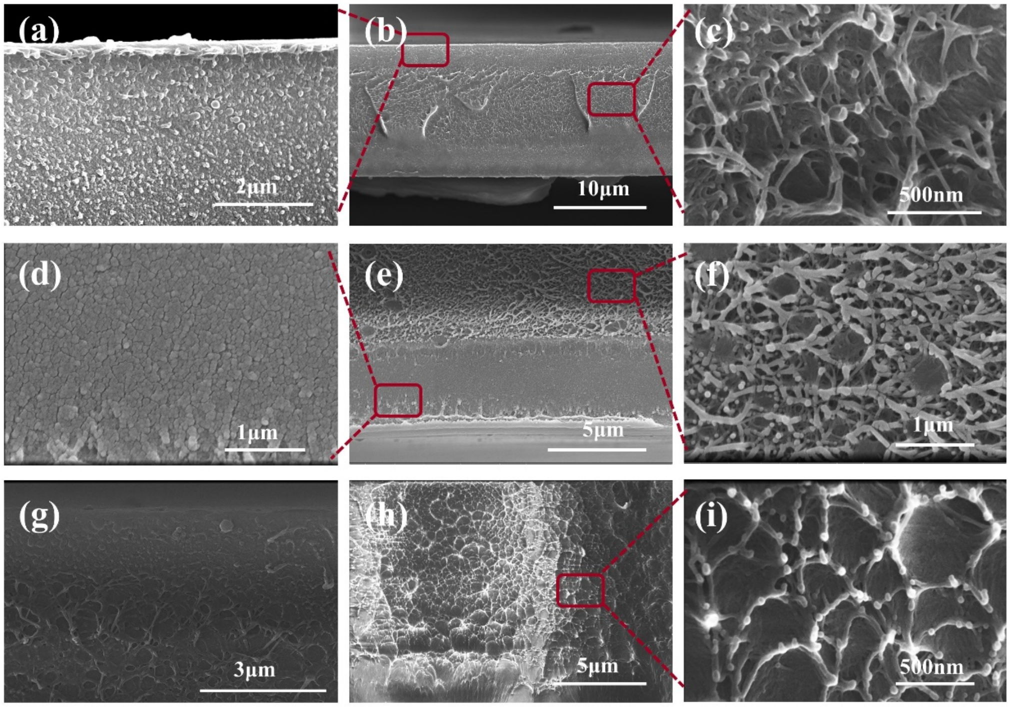

Figure S20. SEM of the as-prepared films: PANI/PEEK films with different thickness. (a-c) 16μm, (d-f) 75 μm, (g-i) 110 μm were were the cross-sectional SEM images of films.

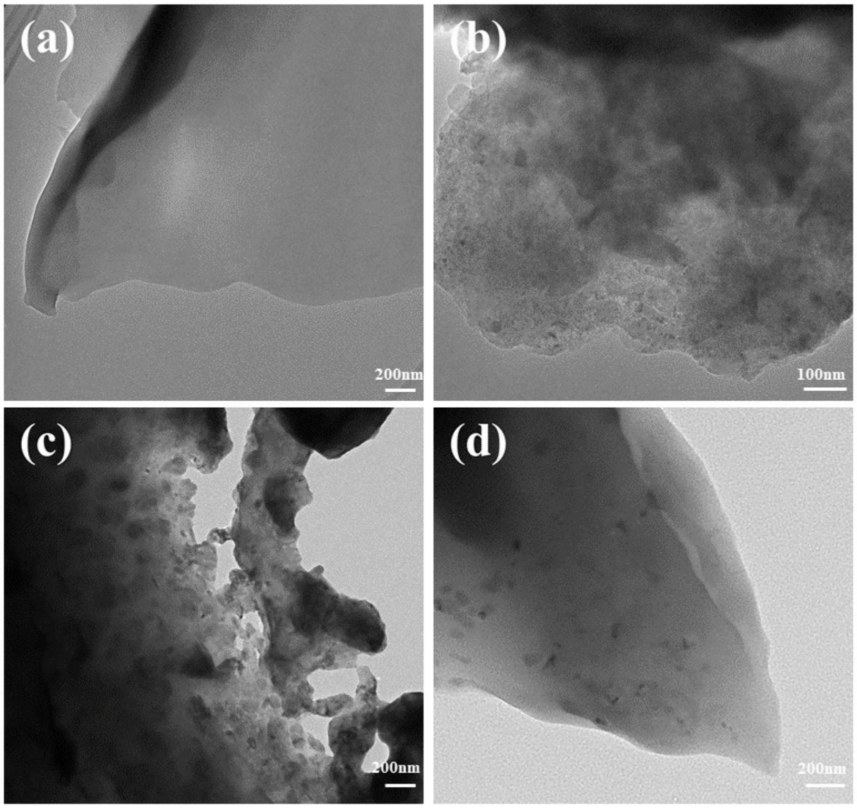

Figure S21. TEM of the as-prepared films: (a)PEEKt film; PANI/PEEK films with different thickness. (b) 16 μm, (c) 75 μm, (d) 120 μm.

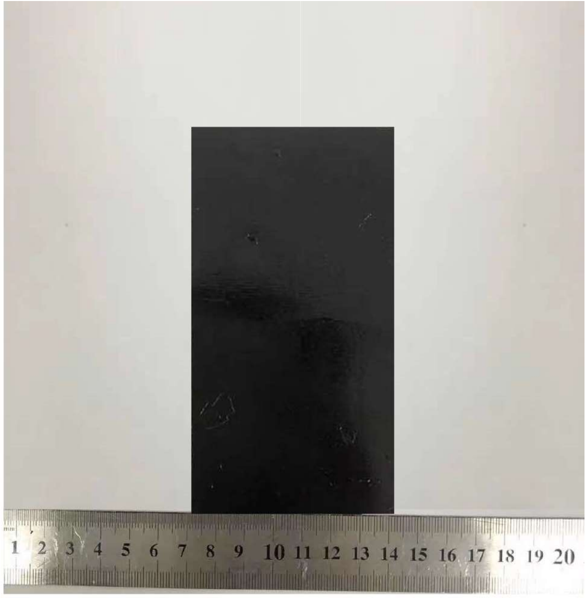

Figure S22. The physical picture of PANI/PEEK composite material which was hot-pressing formed.

6 Supporting Tables

Table S1. The element content of PEEKt film and PANI/PEEK film.

Element content	C (wt %)	H (wt %)	N (wt %)	O (wt %)	Total (wt %)
PEEKt film	81.9	5.6	6.3	6.2	100
PANI/PEEK film	77.2	5.1	5.0	12.7	100

Tables S2. The density (ρ) of PANI/PEEK films with different thickness at room temperature.

Sample	Density (ρ)
PEEKt(HCl) film	1.20
PANI/PEEK film	1.27
Pure PEEK film	1.28
220-PANI/PEEK film	1.29

Table S3. The Tensile Strength (MPa) of PANI/PEEK films with different thickness at room temperature.

Sample	Tensile Strength (MPa)
PEEK	70.8
16 μm-PANI/PEEK	75.8
75 μm-PANI/PEEK	75.0

Table S4-PANI/PEEK films – Temperature (T)

Sample	Temperature (ºC)	c[$(NH_4)_2S_2O_8$] (mol/L)	c(HCl) (mol/L)	Reaction time (h)	Thickness (μm)
T-1	20	0.18	1.0	6	60
T-2	40	0.18	1.0	6	60
T-3	60	0.18	1.0	6	60
T-4	80	0.18	1.0	6	60
T-5	100	0.18	1.0	6	60
T-6	120	0.18	1.0	6	60
T-7	140	0.18	1.0	6	60
T-8	160	0.18	1.0	6	60
T-9	180	0.18	1.0	6	60
T-10	200	0.18	1.0	6	60
T-11	220	0.18	1.0	6	60

Table S5-PANI/PEEK films – c(HCl) (H)

Sample	Temperature (ºC)	$c[(NH_4)_2S_2O_8]$ (mol/L)	c(HCl) (mol/L)	Reaction time (h)	Thickness (μm)
H-1	60	0.037	0.5	6	60
H-2	60	0.037	1.0	6	60
H-3	60	0.037	1.5	6	60
H-4	60	0.037	2.0	6	60
H-5	60	0.037	2.5	6	60
H-6	60	0.037	3.0	6	60
H-7	60	0.037	3.5	6	60
H-8	60	0.037	4.0	6	60

Table S6-PANI/PEEK films – c[$(NH_4)_2S_2O_8$] (O)

Sample	Temperature (ºC)	c[$(NH_4)_2S_2O_8$] (mol/L)	c(HCl) (mol/L)	Reaction time (h)	Thickness (μm)
O-1	60	0.009	2.5	6	60
O-2	60	0.018	2.5	6	60
O-3	60	0.027	2.5	6	60
O-4	60	0.037	2.5	6	60
O-5	60	0.046	2.5	6	60
O-6	60	0.055	2.5	6	60

Table S7-PANI/PEEK films – Thickness (μm)

Sample	Temperature (ºC)	$c[(NH_4)_2S_2O_8]$ (mol/L)	c(HCl) (mol/L)	Reaction time (h)	Thickness (μm)
8μm-PANI/PEEK	60	0.037	2.5	6	8
16μm-PANI/PEEK	60	0.037	2.5	6	16
30μm-PANI/PEEK	60	0.037	2.5	6	30
75μm-PANI/PEEK	60	0.037	2.5	6	75
110μm-PANI/PEEK	60	0.037	2.5	6	110

References

[S1]. S.-J. CHEN, D. G. HOWITT, A. B. HARKER, Scanning Vol. **2000**, 22, 156–160.

[S2]. L. Di, Y.J. Xie, S. Li, ACS Appl. Energy Mater. **2019**, 2, 1646−1656.

[S3]. N. Cao, Y. Sun, J. Wang, Chemical Engineering Journal. **2020**, 386, 124086.

[S4]. N. Cao, C. Yue, Z.Y. Lin, Journal of Hazardous Materials. **2021**, 414, 125489.

Author Contribution

Z.Y.L., N.C., Z.H.S., and J.H.P. conceived the concept and designed experiments. Z.Y.L., W.Y.L., and Z.H.S. performed the experiments. Z.Y.L, Z.H.S., Y.R.S. and J.H.P. analyzed the data and discussed the results. Z.Y.L., N.C., Z.H.S. J.H.P., H.B.Z. and Z.H.J. wrote the manuscript.
